# Deficit in feature-based attention following a left thalamic lesion

**DOI:** 10.1016/j.neuropsychologia.2017.05.023

**Published:** 2017-07-28

**Authors:** Sofia Finsterwalder, Nele Demeyere, Celine R. Gillebert

**Affiliations:** a Oxford Cognitive Neuropsychology Centre, Department of Experimental Psychology, University of Oxford, Oxford, United Kingdom; b Laboratory of Experimental Psychology, Department of Brain & Cognition, University of Leuven, Leuven, Belgium

**Keywords:** Feature-based attention, Stroke, Thalamus, Attention weights, Endogenous control

## Abstract

Selective attention enables us to prioritise the processing of relevant over irrelevant information. The model of priority maps with stored attention weights provides a conceptual framework that accounts for the visual prioritisation mechanism of selective attention. According to this model, high attention weights can be assigned to spatial locations, features, or objects. Converging evidence from neuroimaging and neuropsychological studies propose the involvement of thalamic and frontoparietal areas in selective attention. However, it is unclear whether the thalamus is critically involved in generating different types of modulatory signals for attentional selection. The aim of the current study was to investigate feature- and spatial-based selection in stroke survivors with subcortical thalamic and non-thalamic lesions. A single case with a left-hemispheric lesion extending into the thalamus, five cases with right-hemispheric lesions sparing the thalamus and 34 healthy, age-matched controls participated in the study. Participants performed a go/no-go task on task-relevant stimuli, while ignoring simultaneously presented task-irrelevant stimuli. Stimulus relevance was determined by colour or spatial location. The thalamic lesion case was specifically impaired in feature-based selection but not in spatial-based selection, whereas performance of non-thalamic lesion patients was similar to controls’ performance in both types of selective attention. In summary, our thalamic lesion case showed difficulties in computing differential attention weights based on features, but not based on spatial locations. The results suggest that different modulatory signals are generated mediating attentional selection for features versus space in the thalamus.

## Highlights

• Six stroke survivors performed a feature- and spatial-based variant of the SART.
• A left thalamic patient was impaired in feature- but not space-based attention.
• Performance of 5 right non-thalamic patients did not differ from healthy controls.
• Our data suggest feature- and space-based attention are dissociated in the thalamus.

## 1 Introduction

Selective attention is an effective mechanism to cope with the daily flood of sensory information that reaches our senses and typically exceeds the limited processing capacity of our brain. Attention can be directed to spatial locations (Posner, 1980, Posner et al., 1982, Treisman and Gelade, 1980), features such as an object's colour or shape (Baylis et al., 1993, Driver and Baylis, 1989, Harms and Bundesen, 1983, Kramer and Jacobson, 1991, Maunsell and Treue, 2006) as well as whole objects (Duncan, 1984, Egly et al., 1994, Kanwisher and Driver, 1992, Vecera and Farah, 1994). Spatial-, feature- and object-based perceptual representations are weighted depending on the expectations and internal goals of the observer and integrated with information about the perceptual properties of the stimuli (e.g. the physical salience). The computed attention weights can be topographically represented in a priority map reflecting which elements will be preferentially processed (Bisley and Goldberg, 2010, Bundesen et al., 2005; Desimone and Duncan, 1995; Liu et al., 2011).

The concept of priority maps for attentional selection dates back to the concept of saliency maps, which has been introduced by Itti, Koch and colleagues (Itti and Koch, 2001, Koch and Ullman, 1985, Baluch and Itti, 2011). These authors described a computational model of ‘bottom-up’ selection. According to this model, visual input is first filtered by different feature-detection subsystems to create feature maps, e.g. for orientation, luminance and colour. Neural activation in each feature map represents the salience of that feature across the visual field. These feature maps are combined into a single saliency map, whose “peak” determines attentional selection of the target object based on a winner-take-all mechanism (Bisley and Goldberg, 2010, Bundesen et al., 2011). Importantly, in this model, ‘saliency maps’ are computed in a purely bottom-up manner. We use the term ‘priority map’ to emphasise bottom-up and top-down influences on attentional selection.

Converging evidence from functional neuroimaging and lesion-based studies indicate that modulatory signals mediating attentional selection are generated by a large-scale network of regions in the frontoparietal cortex and the thalamus (for recent reviews, see Ptak, 2012; Scolari et al., 2015; Vandenberghe and Gillebert, 2015; Vandenberghe et al., 2012). Functional neuroimaging studies in healthy volunteers show overlapping activations during spatial-, feature- and object-based attention within the frontoparietal network (e.g., Bressler et al., 2008; Egner et al., 2008; Ester et al., 2016; Fink et al., 1997; Giesbrecht et al., 2003; Hou and Liu, 2012; Liu, 2003; Peelen and Mruczek, 2008; Scolari et al., 2015). For instance, Giesbrecht et al. (2003) found that both colour and location cuing were associated with activity in the superior frontal and posterior parietal cortex. However, besides the common, domain-independent control network, distinct subpopulations of neurons are associated with feature- and spatial-based attention and even with attention to different features (Liu et al., 2011). In particular, neurons in the prefrontal cortex appear to be the source of feature-based attention (Bichot et al., 2015) (Baldauf and Desimone, 2014). Other components of the frontoparietal network are specialised for spatial-based attention, such as the right inferior parietal lobule, which appears to be more responsive to spatial cues than to colour cues (Vandenberghe et al., 2001b). Thus, different regions within the common, frontoparietal control network represent attentional priority to spatial locations, features and objects.

Besides the frontoparietal network, parts of the thalamus have been associated with modulatory signals mediating attentional selection. In a single case study, a patient with a right thalamic lesion including the pulvinar nucleus performed a partial report task, where red target letters have to be reported, while green distractor letters have to be ignored. This patient showed lateralised attentional weighting towards the targets in the ipsilesional field (Habekost and Rostrup, 2006). The authors interpreted the single case findings within the framework of the theory of visual attention (TVA), which states that the topographically organised priority map with stored attention weights is represented in the pulvinar nucleus of the thalamus (Bundesen et al., 2005, Bundesen et al., 2011). In the same study, patients with lesions outside the thalamic area showed a similar impairment as the single case. However, the non-thalamic lesions were large, including the basal ganglia, the frontal cortex and in some cases extended to the temporal and parietal cortices and, therefore, might have incorporated parts of the frontoparietal network, which is associated with feature- and spatial based attention as described above. In a subsequent study, 16 patients with different thalamic lesions to the right or left hemisphere performed the partial report task (Kraft et al., 2015). Patients did not suffer from neglect or other attentional disturbances as shown by standard clinical testing. The left pulvinar damage of one patient replicated the finding of a spatial attention bias to the ipsilesional side. Medial thalamic damage in 9 of the patients was related to biased attention weights either to the ipsi- or to the contralesional side and the remaining lateral thalamic lesion patients showed a deficit in processing speed and no bias of weights (Kraft et al., 2015).

Further support for attentional weighting bias in patients with pulvinar lesions comes from a study by Snow et al. (2009). Participants were asked to discriminate the orientation of a lateralised target grating in the presence of distractors with varying salience. Compared to controls, patients were impaired in discriminating target features, but only if targets were presented together with highly salient distractors. The deficit was stronger in the contralesional than in the ipsilesional field. The perceptual salience of the distractors competed with the behavioural relevance of the target. The results suggest that the pulvinar plays an important role in filtering irrelevant but salient distractors by representing high attention weights for targets and low weights for distractors. Snow and colleagues (2009) point out that the thalamus is reciprocally connected to the frontoparietal network and visual areas. They argue that the frontoparietal network coordinates attentional selection signals in visual areas via the thalamus. Thus, damage to the pulvinar disrupts the coordination of attentional feedback signals resulting in contralesional impairment in filtering salient distractors (Snow et al., 2009, Strumpf et al., 2013). Analogously, a study in non-human primates underlines the critical role of the pulvinar in attentional selection. The macaques’ performance in colour discrimination at a cued location was impaired in the presence of a distractor when the pulvinar had been unilaterally deactivated via muscimol injections. In contrast, the target colour was correctly identified in trials without competing distractors (Desimone et al., 1990).

In sum, the reviewed evidence suggests that parts of the thalamus generate modulatory signals mediating attentional selection. This has been tested using paradigms that rely on a combination of feature- (e.g. colour or orientation) and spatial-based attention. However, the role of the thalamus in solely feature-based or spatial-based attention has not specifically been addressed yet. It remains unclear, whether the thalamus is involved in different types of selective attention as has been demonstrated for the frontoparietal network.

The aim of the current study was to investigate feature- and spatial-based attention in stroke patients with thalamic and non-thalamic lesions. To this end, we applied a variant of the Sustained Attention to Response Task (SART; original version by Robertson et al., 1997), a go/no-go task based on a feature (i.e. colour) or a spatial selection criterion of relevant stimuli. Participants were asked to respond to task-relevant stimuli, while ignoring simultaneously presented task-irrelevant stimuli (i.e. distractors), which could be highly salient or low salient. Subjects with high abilities of feature- and spatial-based attention should be less influenced by the presence of a distractor and show similar performance in low and highly salient distractor trials. Thus, the task requires sustained attention across trials and selective attention to select the relevant stimuli.

Our SART variant differs from the standard approach to investigate feature-based selective attention. Most findings are based on tasks, where one or several targets are presented among multiple distractors throughout the visual field, e.g. visual search tasks (e.g., Gillebert et al., 2012). Hence, a combination of spatial- and feature-based attention is necessary for attentional selection (e.g., Baldassi and Verghese, 2005; Egner et al., 2008; Malhotra et al., 2009; White et al., 2015). The new SART version was designed to disentangle feature-based and spatial-based attention while maintaining the same stimulus type and to avoid a potential confound of both selection types. Two stimuli are shown at once and are either overlapping (separable by colour) or spatially apart (separable by location). Also, the new SART variant is a selective attention task against a sustained attention baseline (Liu, 2003, Molenberghs et al., 2007, Shomstein and Yantis, 2004, Vandenberghe et al., 2001a, Yantis et al., 2002). Most of the prior research on spatial- or feature-based attention made use of cueing tasks or whole/partial report tasks in which trials are separated by a fixed or random intertrial interval. However, in everyday life, the attentional priority map is *continuously* updated based on changes in the spatial location, the features, the sensory salience and the behavioural relevance of stimuli in the environment. The new SART variant enabled us to study the dynamic calibration of attentional priorities, while controlling for processes related to maintaining attention over time.

## 2 Methods

The study was approved by the Medical Science Interdivisional Research Ethics Committee of the University of Oxford (MSD-IDREC-C1-2013-41). All participants gave written informed consent in accordance with the Declaration of Helsinki.

### 2.1 Participants

Six patients with subcortical ischaemic lesions were consecutively recruited via the Oxford Cognitive Neuropsychology Centre of the University of Oxford. Extension into the insula and inferior frontal cortex was permitted based on the known distribution of the vascular territory of the posterior branches of the middle cerebral artery. Exclusion criteria were age above 85 years, pre-existing structural lesions or extensive periventricular or subcortical white matter hyperintensities, presence of hemianopia, colour blindness, insufficient balance to sit autonomously in front of a computer and general inability to understand and perform a computerized perceptual discrimination task. Patients were tested during the chronic phase of the stroke (mean time post-stroke 941 days, standard deviation 229 days). One patient (Case 1) had a lesion extending into the left thalamus, the other patients (Cases 2–6) had non-thalamic lesions in the right hemisphere. Demographic data of the patients are presented in Table 1. In addition, 34 cognitively intact participants age-matched to the patient with thalamic brain damage (mean age 44.7 years, SD 19.7; 17 female) were recruited via the Oxford Cognitive Neuropsychology Centre pool of volunteers or the wider Oxford Psychology Research Participant Recruitment scheme and reimbursed for their participation (£10 per hour).

**Table 1:** Demographical data.

Patient ID	Age	Sex	Level of education	Time since stroke (in days)	Lesion site	Lesion volume (in cm^3^)
Case 1	45	f	11	877	left	3.1
Case 2	70	m	18	999	right	3.5
Case 3	80	m	11	934	right	21.6
Case 4	67	m	10	837	right	68.5
Case 5	68	m	11	655	right	33.0
Case 6	61	m	13	1346	right	24.5

### 2.2 Neuropsychological screening

The cognitive profile of each patient was derived 6 months post-stroke using the Birmingham Cognitive Screen (BCoS; Humphreys et al., 2012), an extensive cognitive screen designed to detect cognitive impairments in different domains, including memory, language, attention and executive functioning, praxis and number processing (Table 2).

**Table 2:** Neuropsychological profile on the Birmingham Cognitive Screen (BCoS).

**DOMAIN**	**Subdomain**	**Function**	**RANGE**	**Case 1**	**Case 2**	**Case 3**	**Case 4**	**Case 5**	**Case 6**
**MEMORY**	**Orientation**	Personal	0–8	8	8	8	8	8	8
		Time & Space	0–6	6	6	6	6	6	6

	**Long term**	Recall	0–15	6	13	11	3.5	13	10
		Recognition	0–15	13	15	15	12	15	15

	**Short term**	Recall	0–15	3	11	11	–	10.5	7.4
		Recognition	0–15	11	15	14	12	15	14
	**Episodic**	Task recognition	0–10	8	10	10	10	10	10

**LANGUAGE**	**Spoken**	Picture naming	0–14	14	14	13	11	14	14
		Reconstruction	0–8	7	8	8	8	8	8
	**Comprehension**	Comprehension	1–3	–	3	3	3	3	3

	**Writing**	Sentence reading	0–42	42	42	42	41	42	42
		Non-word reading	0–6	5	6	6	3	6	6
		Writing	0–5	–	5	5	0	5	5

**ATTENTION**	**Spatial neglect**	Overall	0–50	49	41	45	45	47	43
		Page Asymmetry	0–20	0	2	1	2	2	2
		Object Asymmetry	0–50	1	0	0	1	0	0

	**Spatial extinction**	Visual - Left space	0–8	8	8	8	8	8	8
		Visual - Right space	0–8	8	8	8	8	8	8
		Tactile - Left space	0–8	8	8	8	8	8	8
		Tactile - Right Space	0–8	8	8	8	8	8	8

	**Executive function**	Rule accuracy	0–18	2	11	10	–	12	11
		Total rules	0–3	1	3	3	–	3	2

	**Auditory attention**	Total accuracy	0–54	52	53	47	53	53	53
		Working memory	0–3	3	3	2	3	3	3
		Sustained attention		0	1	0	0	1	1

**PRAXIS**	**Action**	Object use	0–12	12	11	12	12	11	12
		Gesture production	0–12	12	12	12	12	12	12
		Gesture recognition	0–6	5	6	6	6	6	5
		Imitation	0–12	12	12	12	12	11	11
		Figure copy	0–47	–	45	43	36	44	44

**NUMBER**		Reading	0–9	9	9	9	8	9	9
		Writing	0–5	–	5	5	3	5	4
		Calculation	0–4	3	4	4	1	4	4

*Note:* Underlined values indicate worse performance compared to the norms (Humphreys et al., 2012); - = data not available

### 2.3 Lesion analysis

**Fig. 1:**
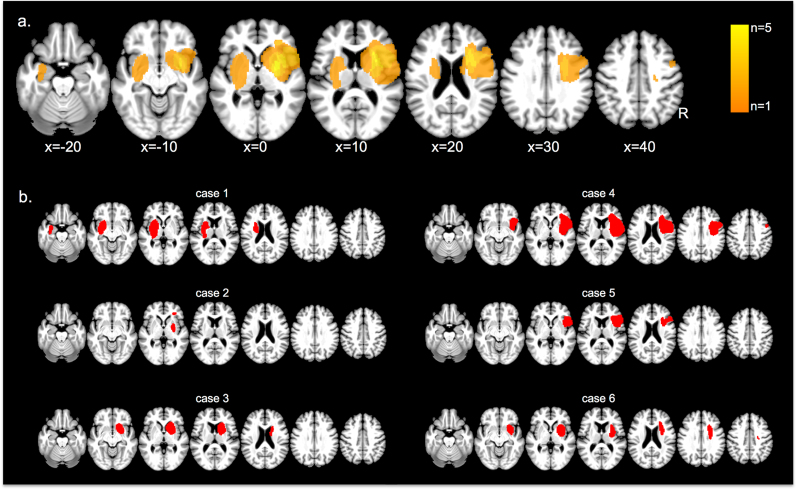
Lesion distribution. (a) Lesion overlay. The colour code indicates in how many individuals of our sample (n = 6) a given voxel was lesioned. (b) Overview of the location and extent of the individual lesions.

All six patients included in the study had unilateral lesions as a result of ischaemic stroke demonstrated by magnetic resonance (n = 4) or computerized tomography scans (n = 2). Magnetic resonance images (MRI) were acquired on a 3 T TIM Trio scanner at the Oxford Centre for Clinical Magnetic Resonance Research (OCMR). For each patient, we acquired a high-resolution 3D whole-brain T1-weighted MRI scans using a magnetization-prepared rapid gradient echo sequence (MPRAGE) (repetition time 3000 ms, echo time 4.7 ms, flip angle 8 degrees, 1 mm isotropic resolution) and a fluid rapid attenuated inversion recovery (FLAIR) image (repetition time = 5000 ms, echo time = 397, in-plane resolution 1 mm, slice thickness 1.5 mm). Two patients were excluded from the MRI data acquisition because of claustrophobia. The boundary of the lesion was delineated on the individual FLAIR image (n = 4, cases 2, 4, 5 and 6) or CT image (n = 2, cases 1 and 3) for every transverse slice with MRIcron (Rorden et al., 2007) and a graphics tablet (Wacom Intuos Pro Medium, Vancouver, Washington, USA). Subsequently, both the anatomical images and the lesions were mapped onto stereotaxic space using the “Clinical Toolbox” (Rorden et al., 2012, https://www.nitrc.org/projects/clinicaltbx/) implemented in the Statistical Parametric Mapping 8 (SPM8) software package (Wellcome Department of Cognitive Neuroscience, University College London, UK; http://www.fil.ion.ucl.ac.uk). The lesion distribution is shown in Fig. 1.

**Fig. 2:**
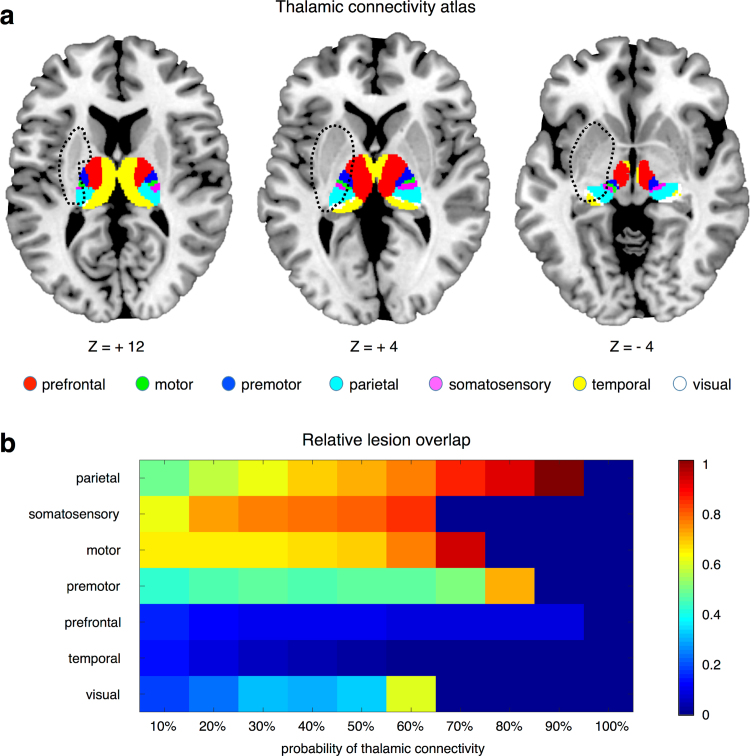
Analysis of thalamic lesion overlap for Case 1. (a) The 7 sub-thalamic regions included in the Oxford Thalamic Connectivity atlas by Behrens and colleagues (Behrens et al., 2003, Johansen-Berg et al., 2005), and the lesion of Case 1 (dotted lines), visualized on axial slices of the brain. (b) We assessed the ratio between the number of lesion voxels in each sub-thalamic region and the total number of voxels in the same region. The analysis was conducted by thresholding the probability maps of sub-thalamic region at different levels, ranging from 10% to 100%.

To characterise the lesion of the thalamic patient (Case 1), we compared it to the Oxford Thalamic Connectivity atlas by Behrens and colleagues (Behrens et al., 2003, Johansen-Berg et al., 2005), as implemented in the SPM Anatomy toolbox (Eickhoff et al., 2005) (http://www.fz-juelich.de/inm/inm-1/DE/Forschung/_docs/SPMAnatomyToolbox/SPMAnatomyToolbox_node.html). The Oxford Thalamic Connectivity Atlas is a probabilistic atlas of 7 sub-thalamic regions, segmented according to their white-matter connectivity to cortical areas (posterior parietal cortex, somatosensory cortex, motor cortex, premotor cortex, prefrontal cortex, temporal cortex, occipital cortex) (Fig. 2a). We defined to what extent each sub-thalamic region, as thresholded at different probability levels (between 10% and 100% in steps of 10%), was lesioned in the patient. This revealed that the patient's lesion had the largest overlap with the thalamic sub-region that is structurally connected to the posterior parietal cortex, followed by the somatosensory- and motor-related parts of the thalamus (Fig. 2b).

## 3 Stimuli and procedure

### 3.1 Apparatus

Stimuli were created and presented through Presentation software (17.0, Neurobehavioural Systems Inc., Berkely, CA, USA, www.neurobs.com), run on a Dell Optiplex 990 computer with a 23-in. ViewSonic VA2342-LED screen (resolution 1920 × 1080 pixels, refresh rate 100 Hz). Participants were seated in a dimly lit room, approximately 70 cm away from the monitor. Responses were collected using a standard keyboard.

### 3.2 Stimuli and task

The experiment consisted of a feature-based and a spatial-based Sustained Attention to Response Task (SART). Participants were presented with two streams of digits. They were instructed to attend to one digit stream (selective attention condition) or both streams (divided attention condition) and respond as quickly as they could to each presented digit pair by pressing the spacebar on the keyboard with their dominant hand, but to withhold the response when a “3” was presented in the attended stream(s).

**Fig. 3:**
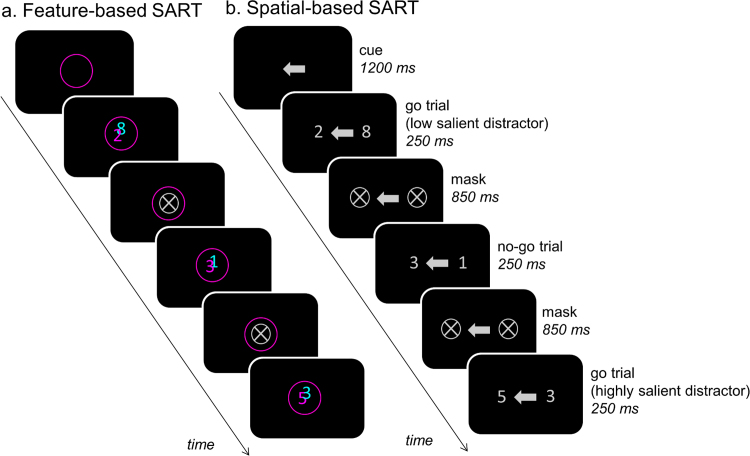
SART schematic: Each block started with a 1200 ms cue presentation indicating the relevant stream (i.e. single-lined ring or single arrow in the single condition) or streams (double-lined ring or double arrow in the double condition). The cue remained visible during the whole block, i.e. two random sequence repetitions of the numbers 1 through 9. Each digit pair was presented for 250 ms. Participants were asked to respond to each pair by pressing the space bar on the keyboard with their dominant hand, but to withhold their response, when a “3” was presented in the relevant stream or streams respectively. Each digit pair was followed by a mask, before a new pair was shown. For instance, in the single magenta (or left) condition, the first number pair depictures a go trial with a low salient distractor (no “3” present), the second a no-go trial (magenta or left “3” present) and the third a go trial with a highly salient distractor (cyan or right “3” present). The digit sequence 1 through 9 was randomised in each stream and all nine numbers were shown before the next sequence started. In the feature task, the numbers were partly overlapping, so that attention to location could not be used to separate the objects. Their presentation in the front or the back randomly changed trial-wise.

Each task followed the same basic design (Fig. 3). Stimuli appeared superimposed against a uniform grey background (RGB: 128, 128, 128). On each trial, we simultaneously presented two equiluminant digits (size: 1.0 cm) for 250 ms followed by a mask for 850 ms. The mask consisted of a light grey ring (RGB: 204, 204, 204; diameter: 2.0 cm) with a diagonal cross in the middle. Stimuli were presented over a total period of ~6 min (324 trials), divided into 18 blocks (total block duration: 19.8 s). Each block contained 18 trials and was preceded by a cue (duration: 1.2 s) instructing participants to attend to one or both streams. The cue remained on the screen for the duration of the block. A block contained twice the digit sequence 1–9 in a random order in each stream, with the constraints that two simultaneously presented digits were never identical. This resulted in five different conditions, as listed in Table 3. In the “selective attention” conditions, participants were asked to attend to the relevant digit stream and ignore the irrelevant digit stream. Task-relevant digits contained a go signal (digits: 12456789) or a no-go signal (digit: 3). Task-irrelevant digits were low salient (digits: 12456789) or highly salient (digit: 3). In the “divided attention” condition, participants were asked to monitor both streams simultaneously and withhold the response if one of the streams contained the no-go digit “3”.

**Table 3:** Experimental conditions and between-group comparisons for the feature-based and spatial-based version of the task.

Condition ID	N	Attention	Target	Distractor	Outcome measure	Thalamic cases vs. healthy controls‡		Thalamic case vs. non-thalamic patients#		Non-thalamic patients versus healthy controls§	
						feature	spatial	feature	spatial	feature	spatial
1	168	selective	go	low salient	misses	p = .15	p = .43	p = .46	p =.26	p = .34	p = .16
2	24	selective	go	high salient	misses	**p = .0002**	p =. 30	**p =.004**	p = .17	p = .71	p = .85
3	24	selective	no-go	low salient	false alarms	p = .15	p = .49	p =.09	p = .26	p = .08	p = .30
4	96	divided	go	n/a	misses	p = .04	p = .35	p = .23	p = .24	p = .51	p = .02
5	12	divided	no-go	n/a	false alarms	p = .38	p = .41	p = .23	p = .31	p = .12	p = .08

*Footnote.* Values that are significant after correcting for multiple comparisons are underlined and in bold.

‡ Case 1 was compared to 34 age-matched healthy controls using a modified *t*-test (Crawford and Garthwaite, 2002).

# Case 1 was compared to five control patients whose right-sided lesion did not extend into the thalamus using a modified *t*-test (Crawford and Garthwaite, 2002).

§ The five control patients were compared to 14 age-matched healthy controls using a non-parametric Kruskal-Wallis test.

In the feature-based SART, two overlapping streams of coloured digits were presented in the centre of the screen (Fig. 3a). Each stimulus pair contained a cyan digit and a magenta digit. The digits were partially overlapping so that attention to location could not be used to separate the stimuli and thus allow the effects to be validly attributed to feature-based attention. We counterbalanced which colour was presented slightly to the left and to the right, and which colour was presented in front or in the back. The stimuli were surrounded by a coloured ring (diameter: 1.6°, ~ 2 cm) instructing participants to attend the cyan colour, the magenta colour or both colours simultaneously.

In the spatial-based SART, two streams of black digits were presented to the left and the right of the fovea on the horizontal meridian (Fig. 3b). An arrow (size: 1.5 cm) pointing to the left, the right or in both directions was presented at the fovea, instructing participants to attend to the left location, the right location, or both locations simultaneously.

Subjects were instructed to fixate the centre of the screen throughout the whole experiment, to avoid eye movements and to attend the digits in the periphery of their vision only. They were asked to give equal importance to accuracy and speed while doing the task. Each subject started with a practice run consisting of one block for each condition. The order of the tasks was counterbalanced across participants.

Gaze fixation was monitored online by means of infrared eye monitoring (Tobii X2-30 Eye Tracker; Tobii Pro) throughout the training and task.

### 3.3 Analysis of behavioural data

Performance of feature- and spatial-based attention was investigated by measuring the percentage of misses on go trials in conditions 1, 2 and 4 and the percentage of false alarms on no-go trials in conditions 3 and 5 (see Table 3). The primary outcome analysis comprised the contrast between go trials with a highly versus low salient distractor (“competition effect”: condition 2 versus condition 1) reflecting the ability to selectively attend the relevant stream while ignoring the irrelevant stream. Low salient distractor trials (condition 1) served as a sustained attention baseline. The contrast allowed separating effects of selective attention from effects of sustained attention (see, for instance, Molenberghs et al., 2007). The primary outcome analysis further included the contrast between divided and selective attention in go and no-go trials (“divided attention effect”: condition 4 versus condition 1, condition 5 versus condition 2) reflecting the ability to divide attention across different features and locations. Inferences between the individual patient with a left thalamic lesion and healthy controls as well as the patients with right non-thalamic lesions were based on multiple modified *t*-tests (Crawford and Garthwaite, 2002). Performance of the 5 cases with non-thalamic lesions was compared to performance of a subset of the healthy controls matched in age (n = 14, mean age 67 years, standard deviation 8, 9 female) using the non-parametric Kruskal-Wallis test. The significance level was Bonferroni-corrected and set to a corrected *p*-value < .05.

In a secondary analysis, we compared the outcome variables for each of the 5 conditions between the three groups (significance threshold: Bonferroni corrected *p*-value < .05, uncorrected *p—*value < .01). To rule out speed-accuracy trade-offs (Seli et al., 2012, Seli et al., 2013), we repeated the statistical tests using reaction time (RT) and RT variability (standard deviation / mean) as outcome variables.

Given the laterality of the lesions, we also explored whether performance on the spatial-based SART differed depending on the spatial location (contra- versus ipsilesional) of the relevant stimulus stream. To this end, we contrasted the percentage of misses (conditions 1 and 2) and false alarms (condition 3) for cued-left versus cued-right selective attention conditions. The significance level was Bonferroni-corrected and set to a corrected *p*-value < .05.

## 4 Results

### 4.1 Misses on go trials

**Fig. 4:**
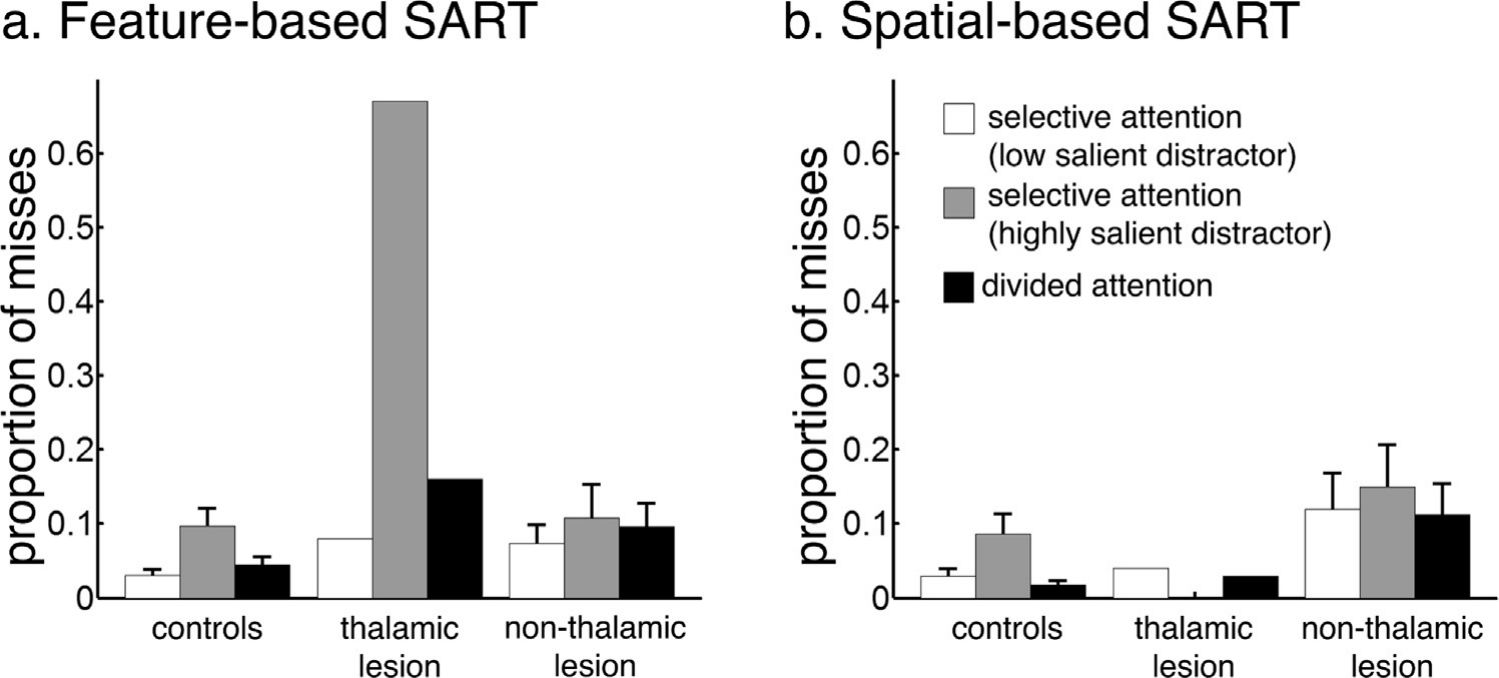
Proportion of misses on go trials made by controls (n = 34), the single thalamic-lesion case (n = 1) and non-thalamic lesion patients (n = 5) during a) the feature-based SART and b) the spatial-based SART. The difference between the grey bar (condition 2) and the white bar (condition 1) reflects the competition effect. The difference between the black bar (condition 4) and the white bar (condition 1) reflects the divided attention effect. Error bars represent *SEM.*

Fig. 4 shows the percentage of misses made by each group and the single thalamic lesion case during the feature-based and the spatial-based task.

In the feature-based task, the single patient with a thalamic lesion did not differ from age-matched healthy controls in the percentage of misses on go trials with a low salient distractor (Table 3). However, the patient showed a pathological increase in the percentage of misses in the contrast between low salient distractor trials and highly salient distractor trials (competition effect: *t* = 4.22, *p* < .0001) (Fig. 4a, difference between grey bar and white bar). In addition, compared to age-matched controls the patient showed a stronger increase in the percentage of misses when two streams instead of one stream had to be monitored (divided attention effect: *t* = 2.39, *p* = .01) (Fig. 4a, difference between black bar and white bar). Similar results were obtained when comparing the single case to patients with a right-sided non-thalamic lesion (competition effect: t = 7.67, *p* < .0001; divided attention effect: t = 2.05, p = .055). Patients with right-sided non-thalamic lesions did not significantly differ from 14 age-matched healthy controls at baseline (Table 3), in the competition effect (χ(1) = .86, p = .34) or the divided attention effect (χ(1) = .21, p = .64). Even at the single-case level, none of the cases differed significantly from controls on the competition or divided attention effect *(*all *p*s *>* .05).

In the spatial-based task, the left thalamic lesion case did not differ from age-matched healthy controls or from the control patients in the percentage of misses at baseline in low salient distractor trials (Table 3, Fig. 4b). The patient neither differed from controls in the competition effect (compared to healthy controls: *t* = −.71, *p* = .24; compared to control patients: t = −.78, p = .24) nor in the divided attention effect (compared to healthy controls: *t* = .07, *p* = .47; compared to control patients: t = .08, *p* = .47). Compared to 14 age-matched controls, patients with right-sided non-thalamic lesions did not make significantly more misses at baseline (Table 3), and did not show a pathological increase in the percentage of misses when the contralateral distractor was highly salient (χ(1) = .62, p = .43) or when attention had to be divided across two locations (χ(1) = .62, p = .43).

The patients did not show significantly increased reaction times (Supplementary Table 1) or increased reaction time variability (Supplementary Table 2) compared to age-matched controls for any of the conditions in the feature-based or spatial-based SART. No hemifield differences were observed in the spatial-based SART (Supplementary Table 3).

### 4.2 False alarms on no-go trials

**Fig. 5:**
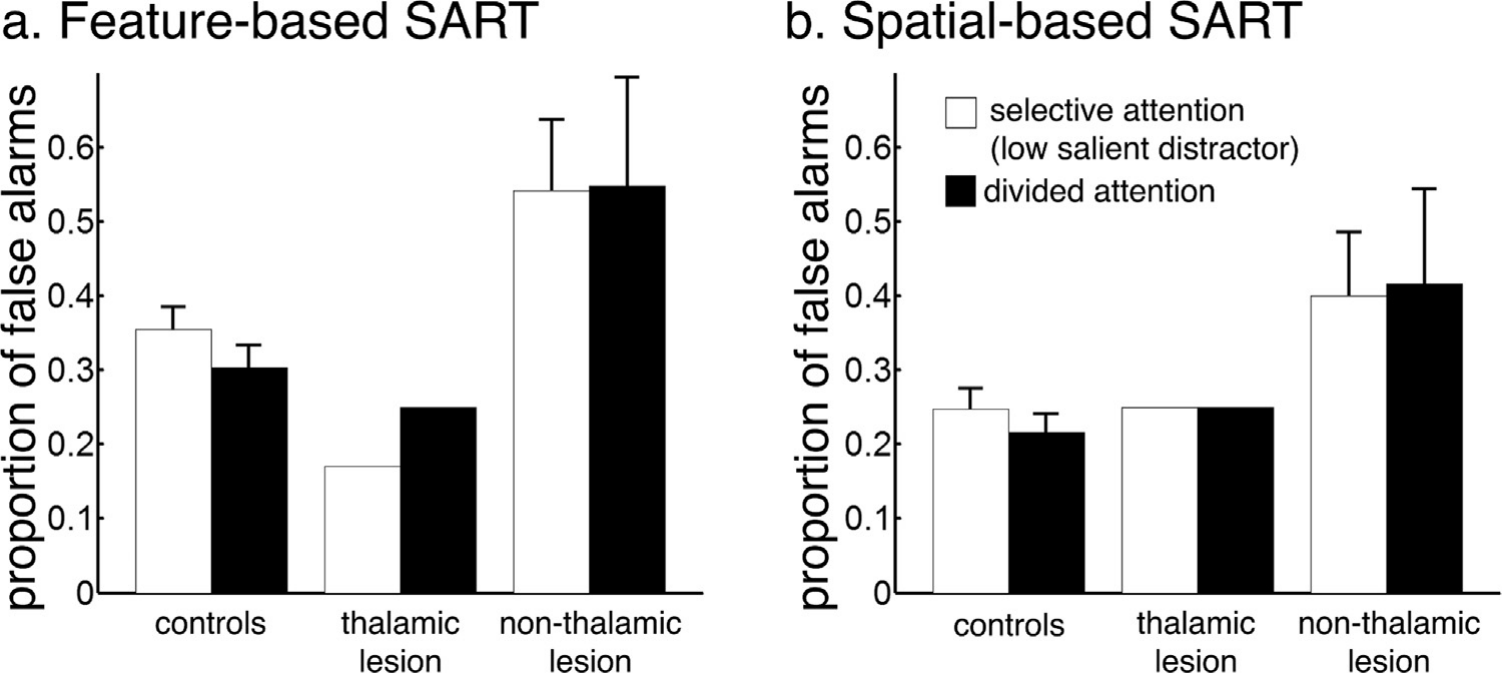
Proportion of false alarms on no-go trials made by controls (n = 34), the single thalamic-lesion case (n = 1) and non-thalamic lesion patients (n = 5) during a) the feature-based SART and b) the spatial-based SART. The difference between the black bar (condition 5) and the white bar (condition 3) reflects the divided attention effect. Error bars represent *SEM*.

Fig. 5 shows the percentage of false alarms made by each group and the thalamic lesion case during the feature-based and the spatial-based SART.

The thalamic lesion case did not show more false alarms than healthy controls in the baseline condition (Table 3), and no pathological increase in false alarms when both streams were relevant (divided attention effect: feature-based SART: *t* = .77, *p* = .22; spatial-based SART: *t* = .17, *p* = .43) (Fig. 5, difference between black bar and white bar). Similar results were obtained when comparing the case to the patient controls (divided attention effect feature-based SART: t = .26; *p* = .40, divided attention effect spatial-based SART: t = −.06, *p* = .48).

The patient controls with non-thalamic lesions did not differ from age-matched healthy controls at baseline (Table 3), or in the divided attention effect (feature-based SART: χ(1) = 1.45, p = .23; spatial-based SART: χ(1) = 1.94, p = .16).

## 5 Discussion

The aim of the current study was to investigate selective attention in stroke survivors with thalamic and non-thalamic lesions. We applied a new version of the SART, a go/no-go task, involving feature-based attention (stimulus relevance determined by colour) or spatial-based attention (stimulus relevance determined by spatial location). We compared performances of a single patient with a left thalamic lesion and five patients with right subcortical lesions but sparing the thalamus, to a group of healthy, age-matched controls. The results demonstrate that the single case with a left-sided lesion extending into the thalamus was specifically impaired in feature-based selection indicated by a pathological increase in attentional capture by highly salient distractors compared to age-matched controls or patients with a right-sided lesion sparing the thalamus. The patient showed no such increase in the spatial-based task. The patient's specific deficit in the feature domain was also reflected by a stronger increase in the percentage of misses on go trials when two streams instead of one stream had to be monitored. The patient was able to divide attention across different locations, but not across features. In contrast, compared to age-matched healthy controls the performance of the non-thalamic lesion patients was not differentially modulated by the presence of highly salient distracters or by the need to divide attention across different stimulus streams.

### 5.1 Dynamic calibration of attentional priorities

This pattern of deficits can be explained within the framework of the “attentional priority map” (Bisley and Goldberg, 2010, Bundesen et al., 2005; Robert Desimone and Duncan, 1995; Liu et al., 2011; Molenberghs et al., 2007). Within this framework, selective attention is characterised by the ability to assign high attention weights to targets and low attention weights to distractors. The higher the object's weight in the priority map, the higher the likelihood of it being processed when the number of objects exceed the capacity of visual short-term memory (Bundesen, 1991, Bundesen et al., 2005). In our tasks, attention weights for both digits were dependent on the observer's knowledge about the relevant digit location (right or left) or digit feature (cyan or magenta), as well as by the go/no-go signal of the digit. The computed priority signals are stored as weights for each digit on the topographically organised priority map, where the higher weight determines digit selection.

Neuropsychological studies suggest that the priority map is located in the thalamus, which is reciprocally connected to the frontoparietal network and visual areas (Bundesen et al., 2005). Lesions to the right medial thalamus and the pulvinar are associated with attentional weighting bias resulting in spatial prioritisation of stimuli in one hemifield (Habekost and Rostrup, 2006, Kraft et al., 2015). In contrast to previous studies, we investigated changes in the attentional priority map against a sustained attention baseline. We showed for the first time that parts of the thalamus may be important to dynamically adapt attention weights based on features. Our data suggest that the brain area lesioned in the single case may be specifically involved in remapping feature priority signals.

The deficit in feature-based selection became overt contrasting highly salient distractor trials against low salient distractor trials. The thalamic lesion case was not able to assign low attention weights to both distractor types. The digit “3” is a highly salient signal compared to other digits in the SART, because it requires a behavioural change in response routine if presented in the relevant stream. The SART relies on quick responses (i.e. button presses) in most of the trials and withholdings in only ~10% of the trials. The frequent target presentation provokes an automatic and rhythmic response mode, which can only be disrupted if attention is maintained and upcoming no-go signals are continuously monitored. Thus, the no-go digit “3” captures attention by its high relevance even if presented in the irrelevant digit stream. The present study showed that healthy participants and patients with non-thalamic lesions were able to filter no-go signals in the irrelevant stream by connecting its behavioural relevance to the relevant colour (feature-based SART) or location (spatial-based SART). The patient with a thalamic lesion might not have been able to flexibly adjust feature-based priority signals. Consequently, the attention weight for the irrelevant no-go signal was higher than for the feature-defined target resulting in an incorrect withholding. It is important to point out that the thalamic lesion case was not suffering from a general impairment in computing attention weights, since the patient was able to successfully filter highly salient distractors based on spatial selection. To our knowledge, there is no study showing a specific deficit in feature-based selection but not in spatial-based selection following stroke.

In the spatial-based version of the task performance of the left thalamic patients as well as the patient control group did not differ depending on the location of the relevant digit stream. In contrast to previous studies reporting a (subclinical) spatial attention bias even in patients without hemispatial neglect (e.g., Bonato et al., 2010; Gillebert et al., 2011, Habekost T. and Bundesen C. Patient assessment based on a theory of visual attention (TVA): Subtle deficits after a right frontal-subcortical lesion, Neuropsychologia, 41 (9), 1171-1188; Kraft et al., 2015), we made use of a continuous performance task with a sustained attention baseline. Specifically, left- versus- right cued trials were manipulated across blocks and participants did not have to shift attention to the contra- or ipsilesional visual field on a trial-by-trial basis. It is also worth noting that the perceptual and working memory demands of our task were lower compared to previous studies that made use of spatial cueing paradigms (e.g., Gillebert et al., 2011), whole/partial report (e.g., Kraft et al., 2015) or dual task (e.g., Bonato et al., 2010) paradigms. Hence, our spatial-based SART task may not have been sensitive to pick up a subclinical spatial attention bias.

### 5.2 Potential influence of perceptual or memory deficits

The feature-based SART and spatial-based SART not only differed in selection criterion. The feature-based SART presents overlapping digits at the fovea (requiring segregation of shapes), while the digits are presented in the periphery in the spatial SART. As the lesion of Case 1 did not span striatal areas involved in object segregation and the performance of the patient was normal in go trials with a low salient distracter, it is unlikely that this caused the deficit in feature-based selection.

In the patient with a thalamic lesion, neuropsychological testing (BCoS) revealed significant short-term memory deficits during immediate recall and recognition compared to controls. Also, the single case showed difficulties in free recall of information stored in long-term memory, but abilities of delayed recognition were spared. It is possible that these memory deficits might have caused the patient's impairment correctly applying the no-go rule in the SART. Although the single case was able to store and retrieve information about the no-go signal indicated by a small number of false alarms, the patient may have not been able to apply the second part of the task rule in the feature task, i.e. attending the cued coloured stream only. However, if the single case suffered from a deficit in storage and/or retrieval of information about the task rules, performance in the spatial task should have been affected too. Previous lesion studies indicated that isolated thalamic damage can cause memory deficits (Aggleton and Mishkin, 1983, Dagenbach et al., 2001, Kubat-Silman et al., 2002, Van der Werf et al., 2003, Voets et al., 2015), albeit usually affecting working memory, which was intact in our single case according to neuropsychological testing. Also, the cue remained visible throughout each block continuously indicating the relevant colour. Therefore, it is not likely that a memory deficit caused the patient's specific impairment in feature-based selection.

### 5.3 The role of the thalamus in remapping feature-based attention priorities

Lesion delineation of our single case indicated damage to the sub-thalamic region that is structurally connected to the posterior parietal cortex, and to a less extent the region connected to the somatosensory and motor cortices (Behrens et al., 2003, Johansen-Berg et al., 2005). Taking into account that the parietal cortex is crucially involved in spatial-based attention (Corbetta and Shulman, 2011, Shulman et al., 2010), one would expect that a (partial) disruption between the thalamus and parietal cortex results in a spatial attention deficit (e.g., Corbetta et al., 2000; Greenberg et al., 2010; Snow et al., 2009; Yantis et al., 2002). Our single case findings do not support this assumption. Instead the thalamic lesion patient showed biased attentional weighting in the feature-based task. Notably, predictions based on affected thalamic sub-regions should be drawn with caution, since neuroimaging data of our single case were acquired on a CT scanner and the lesion may not be delineated to high precision.

The unavailability of demographical variables such as socio-economic status and pre-morbid IQ also warrants a cautious interpretation of the between-group comparisons. In addition, it should be pointed out that we tested a single patient with a left lesion extending into the thalamus and five cases with non-thalamic lesions in the right hemisphere. It is not clear, whether the performance difference between the single patient with a thalamic lesion and the five patients with a non-thalamic lesion rather reflects a lateralisation effect than a structural effect. Yet, there is no evidence that the left hemisphere or left subcortical areas are specifically involved in feature-based attention or feature selection. Furthermore, the lesion was fairly large in some control patients, including white matter tracts, the basal ganglia and the inferior frontal cortex. Further investigations are warranted to assess the contribution of these structures to feature-based versus spatial-based attention.

### 5.4 Future directions

To shed more light on the neural basis of selective attention, future studies should involve further paradigms that differentiate between feature- and spatial-based attention. The lack of neuropsychological findings on specific deficits in selective attention might be due to the applied paradigms, which usually do not allow direct comparison between feature-based and spatial-based selection. An exception is the cueing task based on spatial or non-spatial attention by Giesbrecht and collegues (2003). The task resembles our SART variant but presents bilateral or overlapping rectangles instead of digits. Participants were asked to indicate the orientation of the task-relevant rectangle. Giesbrecht and colleagues (2003) used the paradigm to measure brain activity within the frontoparietal network during spatial and non-spatial attention. Similar paradigms may be helpful to describe the role of the thalamus in selective attention.

## 6 Conclusion

Our findings are in line with the theory that the thalamus represents a topographically organised priority map with stored attention weights determining preferentially processed elements in the visual field (Bundesen et al., 2011, Habekost, 2015, Zhou et al., 2016). The patient with a lesion extending into the thalamus was specifically impaired in feature-based selection but not in spatial-based selection, whereas performance of patients with non-thalamic lesions was similar to controls’ performance in both types of selective attention. Based on the theory of priority maps we propose that distinct attention weights can be computed mediating attentional selection for features versus space in the thalamus. However, the present study does not clarify where exactly and how putative feature or spatial priority signals are generated. More research is needed to characterise the nature of different attentional priority signals in the thalamus.
