# Supplementary material for: Deficit in feature-based attention following a left thalamic lesion
Source: Neuropsychologia. 2017 Jul 28;102:1–10. doi: 10.1016/j.neuropsychologia.2017.05.023 (PMC5555441; doi:10.1016/j.neuropsychologia.2017.05.023)
Supplement: Supplementary file 1 — Supplementary material [file mmc1.docx]

**Deficit in feature-based attention following a left thalamic lesion:**

**Supplementary Material**

Sofia Finsterwalder^1,2^, Nele Demeyere^1^ & Celine R. Gillebert^1,2^

^1^ Oxford Cognitive Neuropsychology Centre, Department of Experimental Psychology, University of Oxford, Oxford, United Kingdom

^2^ Laboratory of Experimental Psychology, Department of Brain & Cognition, University of Leuven, Leuven, Belgium

**Correspondence should be addressed to**

Celine R. Gillebert

Department of Brain & Cognition

University of Leuven

Tiensestraat 102 – box 3711

B-3000 Leuven

Belgium

e-mail: celine.gillebert@kuleuven.be

*Supplementary Table 1. Between-group comparisons on the reaction times for the feature-based and spatial-based version of the task*

| Condition ID | Thalamic cases vs. healthy controls^‡^ | | Thalamic case vs. non-thalamic patients^#^ | | Non-thalamic patients versus healthy controls^§^ | |
| --- | --- | --- | --- | --- | --- | --- |
|  | feature | spatial | feature | spatial | feature | spatial |
| 1 | p = .03 | p = .20 | p = .21 | p = .38 | p = 1.0 | p = .85 |
| 2 | p = .27 | p = .42 | p = .25 | p = .47 | p = .52 | p = .46 |
| 4 | p = .06 | p = .20 | p = .22 | p = .38 | p = .93 | p = 1.0 |

*Footnote.* Values that are significant after correcting for multiple comparisons are underlined and in bold. ^‡^ Case 1 was compared to 34 age-matched healthy controls using a modified t-test (Crawford & Garthwaite, 2002). ^#^Case 1 was compared to five control patients whose right-sided lesion did not extend into the thalamus using a modified t-test (Crawford & Garthwaite, 2002). ^§^ The five control patients were compared to 14 age-matched healthy controls using a non-parametric Kruskal-Wallis test.

*Supplementary Table 2. Between-group comparisons on the reaction time variability for the feature-based and spatial-based version of the task*

| Condition ID | Thalamic cases vs. healthy controls^‡^ | | Thalamic case vs. non-thalamic patients^#^ | | Non-thalamic patients versus healthy controls^§^ | |
| --- | --- | --- | --- | --- | --- | --- |
|  | feature | spatial | feature | spatial | feature | spatial |
| 1 | p = .049 | p = .12 | p = .31 | p = .29 | p = .05 | p = .52 |
| 2 | p = .16 | p = .23 | p = .30 | p = .31 | p = .41 | p = .71 |
| 4 | p = .06 | p = .10 | p = .31 | p = .47 | **p = .001*** | **p = .007*** |

*Footnote.* Values that are significant after correcting for multiple comparisons are underlined and in bold. ^‡^ Case 1 was compared to 34 age-matched healthy controls using a modified t-test (Crawford & Garthwaite, 2002). ^#^Case 1 was compared to five control patients whose right-sided lesion did not extend into the thalamus using a modified t-test (Crawford & Garthwaite, 2002). ^§^ The five control patients were compared to 14 age-matched healthy controls using a non-parametric Kruskal-Wallis test. *Reaction time variability was lower in patients with a right-sided lesion compared to 14 age-matched healthy controls.

*Supplementary Table 3. Between-group comparisons on hemifield differences in the percentage of misses (Conditions 1 and 2) and the percentage of false alarms (Condition 3) for the spatial-based version of the task.*

| Condition ID | Thalamic cases vs. healthy controls^‡^ | Thalamic case vs. non-thalamic patients^#^ | Non-thalamic patients versus healthy controls^§^ |
| --- | --- | --- | --- |
| 1 | p = .31 | p = .38 | p = .51 |
| 2 | p = .43 | p = .38 | p = .42 |
| 3 | p = .13 | p = .19 | p = .96 |

*Footnote.* Values that are significant after correcting for multiple comparisons are underlined and in bold. ^‡^ Case 1 was compared to 34 age-matched healthy controls using a modified t-test (Crawford & Garthwaite, 2002). ^#^Case 1 was compared to five control patients whose right-sided lesion did not extend into the thalamus using a modified t-test (Crawford & Garthwaite, 2002). ^§^ The five control patients were compared to 14 age-matched healthy controls using a non-parametric Kruskal-Wallis test.
